# Impact of Preoperative Quality of Life and Related Factors on the Development of Surgical Site Infections Following Primary Total Joint Arthroplasty: A Prospective Case-Control Study with a Five-Year Follow-Up

**DOI:** 10.1155/2023/7010219

**Published:** 2023-02-02

**Authors:** Styliani Iliopoulou-Kosmadaki, Argyris C. Hadjimichael, Angelos Kaspiris, Ioanna Lianou, Marina Kalogridaki, Ioannis Trikoupis, Panagiotis Touzopoulos, Emmanuel Velivasakis, Ioannis Sperelakis, Emmanouela Dionysia Laskaratou, Dimitra Melissaridi, Elias Vasiliadis, Georgios Kontakis, Panagiotis J. Papagelopoulos, Olga D. Savvidou

**Affiliations:** ^1^First Department of Orthopaedic Surgery, School of Medicine, National and Kapodistrian University of Athens, “ATTIKON” University General Hospital, Rimini 1, Athens 12462, Greece; ^2^Department of Orthopaedics, St Mary's Hospital, Imperial College Healthcare NHS Trust, Praed Street, W2 1NY, London, UK; ^3^Laboratory of Molecular Pharmacology, School of Health Sciences, University of Patras, Patras 26504, Greece; ^4^Department of Orthopaedic Surgery, “Rion” University Hospital and Medical School, School of Health Sciences, University of Patras, Patras, Greece; ^5^Accident and Emergency Department, “KAT” General Hospital of Athens-NHS, Nikis 2, Athens 14561, Greece; ^6^Department of Orthopaedic Surgery, Ortho Clinic & Surgery Centre, Alexandroupolis, Greece; ^7^Department of Orthopaedic Surgery, University Hospital of Heraklion, Heraklion, Crete, Greece; ^8^Third Department of Orthopaedic Surgery, School of Medicine, National and Kapodistrian University of Athens, “KAT” General Hospital, Nikis 2, Athens 14561, Greece

## Abstract

**Introduction:**

As surgical site infections (SSIs) after joint arthroplasty contribute to increased morbidity and mortality, they require further surgical intervention, prolonged hospitalisation, and antimicrobial treatment. The aim of our study is to examine the association between preoperative quality of life (QoL) and other predictive factors on the development of SSIs after primary arthroplasty.

**Methods:**

This is a prospective study that enrolled 56 patients with hip and knee primary osteoarthritis who underwent joint replacement. Data were collected from January to March 2017, including patient demographic characteristics, comorbidities, laboratory results, and perioperative clinical data. The patients' QoL was evaluated preoperatively by applying the knee injury and osteoarthritis outcome score (KOOS) and the hip disability and osteoarthritis outcome score (HOOS) for total knee replacement (TKR) and total hip replacement (THR), respectively. A 5-year follow-up was conducted to assess the clinical status of the patients.

**Results:**

66.1% of patients underwent TKR, with 4.9 ± 1.2 days of hospitalisation, 16% of them required autologous blood transfusion, while 33.9% of patients were treated with THR, with 5.7 ± 1 days hospitalisation and 36.8 of them required this type of transfusion. 16 patients were diagnosed with SSIs, with the older of them (>65 years old) presenting lower probability (odds ratio: 0.13, 95% CI: 0.03–0.62) requiring treatment with additional antibiotics, while revision surgery was performed in 3 of these cases, following periprosthetic joint infection (PJI). Overall preoperative QoL was not statistically associated with SSIs, but low QoL scores were associated with higher rates of SSIs and increased levels of postoperative pain (*p* = 0.009 < 0.05).

**Conclusions:**

The duration of each operation (>90 min), the length of hospitalisation (>4 days), and the presence of comorbidities including hypothyroidism and recurrent urinary tract infections were associated with a high risk for SSIs following arthroplasties. On the contrary, this study revealed no association between other comorbidities, including heart coronary disease, hypertension, and diabetes mellitus, with close monitoring of plasma glucose and SSIs. Moreover, the younger the patients, the more likely they were to require treatment with antibiotics. Overall, high QoL index scores were mainly accompanied by low rates of postoperative SSIs and pain.

## 1. Introduction

Surgical site infection (SSI) is a devastating nosocomial complication that occurs after primary total hip (THR) and total knee replacement (TKR), decreasing the success rate by increasing the morbidity and mortality of affected patients [1]. Based on the anatomical site of infection, SSIs are divided into three categories: superficial incisional, deep incisional, and organ/joint infection [2]. According to published data, the incidence of SSI accounts for 1.69% after THR and 2.82% after primary TKR and is classified among the most common complications following orthopaedic surgery and causes for revision surgery [3]. However, the rate of SSIs in revision THR increases to 3.68%, which is associated with significantly worse outcomes [3]. In addition, recent data on the incidence of periprosthetic joint infection (PJI) in primary THR and TKR range between 0.3% and 1.9%, respectively, and up to 10% for revised arthroplasties [4]. Therefore, SSIs contribute to severe postoperative complications, such as rehospitalisation, revision surgeries, or a need for additional procedures, including the use of muscular flaps (use of a median gastrocnemius flap to achieve wound healing has proven to be effective for the treatment of prosthetic knee infections) [5–7] and prolonged antibiotic treatment, which might interact with the patient's comorbidities, as in most cases, joint replacements are performed in elderly patients. Eventually, the costs for the prolonged treatment of the abovementioned complications will skyrocket, affecting healthcare system expenses [8]. According to the results of recent studies [9], economic savings and a reduction in infection rates can be achieved with the use of single-use instruments. Finally, the presence of rare diseases, such as alkaptonuria, in patients suffering from osteoarthritis, makes treatment with arthroplasty challenging, considering that no adequate data exist [10].

The prevention of SSIs is crucial and can be achieved by the detection and sufficient management of specific risk factors. Several preoperative risk factors have been described in the international literature, such as the score of the American Society of Anaesthesiologists (ASA score) >2, prolonged operative time >90 minutes, smoking, peripheral vascular disease, increased body mass index (BMI), National Healthcare Safety Network (NHSN) risk index >2, increased Charlson comorbidity index, and necessity for intraoperative blood transfusion [2]. Therefore, the identification of potential preoperative risk factors linked to increased rates of SSIs after arthroplasty is deemed necessary.

The purpose of this study was to highlight the impact of some common risk factors on the presence of SSIs. Some of these factors are patient-depended, including patient age and comorbidities with a high prevalence among patients treated with arthroplasty (diabetes mellitus (DM), coronary artery disease, and cancer and urinary tract infection). Moreover, the relationship between preoperative quality of life, as estimated using the QoL index, autologous or heterologous blood transfusion, the duration of operation and hospitalisation, and SSIs are evaluated, taking account that the length of stay usually depends on the severity of comorbidities and the presence of SSIs. The main aim of our study was to clarify the extent to which patient-related factors affect the evolution of this infection and the relationship between the factors under investigation and the risk for SSIs. This study is original, relevant, and different from other studies because it also estimates preoperative quality of life and assesses its impact on the occurrence of SSIs.

## 2. Materials and Methods

### 2.1. Study Design

This single-center prospective study took place in the Department of Trauma and Orthopaedics of Heraklion University Hospital. Consecutive patients who had undergone TKR or THR from 1 January 2017 to 31 March 2017 were eligible, according to the inclusion criteria described in Table 1. The patients were monitored with a five-year follow-up and data from two of these follow-up examinations were used for this study: the first from the follow-up examination at 6 weeks and the second at 5 years postoperatively. The study was approved by the Institutional Bioethics Board with registration number 1126/3-2-2017, and the research complies with the 1964 Helsinki Declaration and its later amendments.

### 2.2. Surgical Procedures

All patients underwent general or spinal anaesthesia, and all TKR procedures were performed with a tourniquet inflated during the procedure and deflated fully once the components were inserted, using the medial parapatellar approach, with cemented implants. Moreover, THRs were performed with a posterolateral approach using cemented or cementless components, based on clinical criteria, including age, gender, poor bone quality, or distal bone loss as described using the Dorr classification. Intraoperative low-pressure drains were used in all cases. In some of these cases, according to estimated intraoperative blood loss, a postoperative autologous blood salvage system was used, while 1 g tranexamic acid was applied, when this medication was available for intraoperative use and no contraindications existed. The Sangvia® blood salvage system from Astra Tech was used for autologous blood transfusions.

### 2.3. Antibiotic Prophylaxis

Intravenous antibiotic prophylaxis with cefoxitin 2 g and teicoplanin 600 mg was administered 30 minutes preoperatively and once 48 hours postoperatively, based on the hospital protocol according to well-known guidelines, except in cases with contraindications.

### 2.4. Inclusion and Exclusion Criteria

All patients enrolled in this study were diagnosed with idiopathic hip or knee OA according to the criteria of the American College of Rheumatology [11, 12]. Patients suffering from secondary osteoarthritis, haemochromatosis, hip fractures, inflammatory or autoimmune diseases, malignancies, or with intra-articular administration of steroids were excluded. However, patients with controlled diabetes mellitus were included, as they were not considered as immunosuppressed.

### 2.5. Surgical Site Infections (SSIs)

The diagnostic criteria used to identify SSIs were based on the United States Center for Disease Control and Prevention (CDC) definition and were classified into the following three subtypes: (1) superficial incisional; (2) deep soft tissue of the incision; and (3) organ/space infection [13]. Superficial incisional infections occur within 30 days after surgery involving the skin and subcutaneous tissue at the wound site. Superficial incisional infections are characterised by redness, swelling and local pain, purulent discharge, spontaneous wound dehiscence, and positive conventional cultures. Deep soft tissue infections occur usually within 90 days after surgery involving the underlying fascia and deep muscular layers with more severe clinical symptoms, such as fever. Finally, organ/space infection refers to infection within 30 days postoperatively, if no implant is left in place or within one year if implants are left in place. The infection seems to be related to the procedure and may concern any anatomical location other than the incision site.

### 2.6. Preoperative Quality of Life (QoL) Assessment

Patient-reported outcome measures (PROMs) are very valuable questionnaires used as benchmarking tools to examine patients' preoperative quality of life indices, such as pain and functionality [14]. The preoperative QoL among our patients was assessed via the knee injury and osteoarthritis outcome score (KOOS) for patients with TKR and the hip disability and osteoarthritis outcome score (HOOS) for patients with THR [15, 16]. KOOS was chosen among other scores, including (knee society score) KSS, which is used to evaluate the clinical outcome following TKR in various studies, including a retrospective one estimating the use of high-crosslinked polyethylene after TKR or other studies estimating the difference in clinical outcome between TKR retaining and TKR scarifying PCL (posterior cruciate ligament) [17, 18]. Similarly, the validity of the HOOS-PS (physical function shortform) was evaluated based on existing studies and was characterised as problematic due to a lack of relevance, insufficient comprehensiveness, and poor comprehensibility [19]. Finally, all patients consented and completed the QoL assessment score form preoperatively.

### 2.7. Statistical Analysis

Statistical analysis was performed using SPSS (IBM Corp. Released 2012, IBM SPSS Statistics for Windows, Version 23.0. Armonk, NY: IBM Corp). Frequency distributions were calculated and the estimation of relative frequency and the 95% confidence intervals (95% CI) were calculated using bootstrap techniques. For the comparison and the degree of correlation of the variables and the evaluation of sample normality, the binomial test (estimation at 50%), Mann–Whitney U, and chi-square (*χ*^2^) tests were applied. For the control between “preoperative” and “exit” values, a student's paired observation test was performed, while for the correlation of variables, multiple logistic regression analysis was used.

## 3. Results

### 3.1. Sample Characteristics

The demographic characteristics of the patients enrolled in our study included 67.9% women and 32.1% men with a mean age of 69.6 ± 9.8 years (Table 1). In addition, 12.5% of them were smokers, 66.1% diagnosed with hypertension, and 26.8% had ≥3 comorbidities.

### 3.2. Surgery Characteristics

Overall, a consecutive series of 56 patients who fulfilled the inclusion criteria and were estimated with preoperative and postoperative laboratory tests (Tables 2 and 3) was enrolled in the study. 66.1% of them underwent TKR and 33.9% underwent THR (*p* = 0.022), while the operative time and hospital stay for both procedures were calculated (Table 4). Regional spinal anaesthesia was applied in 69.1% (*n* = 38) and general anaesthesia in 30.9% (*n* = 17) of the patients enrolled in the study (*p* = 0.055). An autologous blood transfusion system was used in 36.8% (*n* = 7) of patients who underwent THR and in 16.2% (*n* = 6) of patients who underwent TKR (*p* = 0.104).

### 3.3. Incidence of SSI

28.6% (*n* = 16) of the patients who participated in our study (95% CI [17.9–82.1]) developed SSI. The median duration of antibiotic administration was 17 ± 11.3 days. The most commonly used antibiotic was quinolone in 56.3% (*n* = 9) (95% CI [31.3–81.31]) of SSI cases, followed by penicillin in 25% (*n* = 4) (95% CI [6.3–50]) of cases. Most patients with established SSI complained of pain (48.2%) (95% CI [35.7–62.5]) followed by oedema and swelling at the joint (28.6%) (95% CI [17.9–82.1].

### 3.4. Infective Organisms Identified

In this study, infective microorganisms were identified in 50% of SSIs. The main causative agent was *Staphylococcus* (*n* = 6), corresponding to 37.5% of SSIs, with methicillin-resistant *S. aureus* (MRSA) being isolated in 3 patients, whereas methicillin-sensitive *S. aureus* (MSSA) and coagulase-negative staphylococcus were isolated in 2 and 1 patients, respectively. Moreover, *Enterococcus sp.* (*n* = 1) and *E. coli* (*n* = 1) were also detected.

### 3.5. Risk Factors Associated with SSI

According to the demographics in Table 5, patients aged ≥65 years received antibiotic treatment for SSI at a statistically significant lower rate of 87% (odds ratio: 0.13, 95% CI:0.03–0.62) compared to younger patients. Consequently, patients aged ≤65 treated with THR or TKR appeared at higher risk (55.6%) for SSI compared to elderly individuals (15.8%), which was not related to demographic or surgical characteristics (chi-square test: *p*=0.002). Moreover, the relationship between other factors under investigation and SSIs is depicted in Table 5.

### 3.6. Quality of Life

The preoperative assessment of QoL in patients with THR or TKR is displayed in Table 6. Based on the preoperative assessment results, 16 out of 56 patients with SSI had a significantly lower QOL questionnaire score and increased pain levels (33.9 vs 45.7, respectively, *p*=0.009). However, no other statistically significant differences were noted (*p* > 0.05) (Table 7).

### 3.7. Follow-Up

During the 5-year follow-up, 3 of the patients diagnosed with SSIs developed a periprosthetic joint infection, and revision surgery was performed. One of the patients enrolled died, with the cause of death not related to the procedure under investigation.

## 4. Discussion

The aim of this study is to identify the correlation between the development of SSIs following THR or TKR and possible risk factors. The patient's preoperative QoL level is an interesting and clinically relevant factor, considering that there is no published data evaluating the association between this index and SSIs following arthroplasty. According to the results, patients with SSIs experienced more disabling symptoms preoperatively, such as joint pain, reduced joint function, and more discomfort compared to those with aseptic THR and TKR. Symptomatic osteoarthritis is a cause of chronic stress because patients have difficulty in performing daily activities and may be socially isolated because of their disability to move, resulting in a low score in the QoL questionnaire. Indeed, increased chronic stress promotes a proinflammatory phenotype characterised by the ascendance of immune cells and elevated production of cytokines, accompanied by a reduction of inhibitory signals [20]. Additionally, recent experimental data in humans and animal models has shown that stress induces a hyperinflammatory state in myeloid cells that is accompanied by alterations in chromatin structure at inflammatory and metabolic gene loci, elevated expression of inflammatory genes, and increased cytokine secretion [21]. It can be considered that the increased incidence of SSIs following THR or TKR in patients with poor preoperative QoL indices are linked to low-grade inflammatory reactions caused by chronic physical or mental stress [22].

The incidence of SSIs among the subjects included in this study was higher (28.5%) compared to those of other studies including: Sane and Samant (6.84%) [23], Jegiasi et al. (12.3%) [24], Jain et al. (19%) [25], but similar to Maksimovic with a reported SSI frequency of 22.7% [25]. Additionally, the incidence of SSIs after elective procedures in the elderly was only 1.5% [26], while according to Marusic et al. [2], it was 5.4 and 4.8 for THA and TKA, respectively. Moreover, large cohort studies from the USA reported very low rates of SSIs, around 2.3% [27], and UK research studies reported an SSI incidence of 2.23% following primary THA and 4.97% following hemiarthroplasty [28]. This range of incidence values relates to the inclusion and exclusion criteria of these studies, the type of wounds under examination, and the factors affecting infection monitoring. Moreover, the incidence of SSI depends on the duration of follow-up, as more than 35% of SSIs were detected after hospital discharge, leading to cases of SSIs not being diagnosed [27]. Consequently, close monitoring for long periods is recommended [27].

*S. aureus* and coagulase-negative staphylococci were the most prevalent causative microbes, corresponding to 75% of infectious agents, which was consistent with the results of other studies, revealing that MRSA and MSSA were the most common causative microorganisms, followed by coagulase-negative staphylococcus, *Enterococcus sp.*, *Pseudomonas aeruginosa,* and Proteus [2, 12, 26–29], given that staphylococci are part of the normal flora of the skin and are associated with nasal colonisation [30]. However, no pathogenic microorganisms were identified in laboratory blood tests in cultures from joint or wound aspirations in 50% of patients. In accordance with the published data, many periprosthetic joint infections are misdiagnosed as 20–30% of conventional cultures appear false negative [31], as early SSIs are caused by more virulent pathogenic microorganisms such as *Staphylococcus aureus* or enteric gram-negative bacilli (e.g., *E. coli*) and delayed SSIs by less aggressive pathogens such as coagulase-negative staphylococci [32]. According to Lewis et al. the diagnosis of SSIs in patients undergoing THR tends to be earlier (approximately 23 days) compared to those with TKR (33 days) [32]. Close postoperative monitoring seems to affect prompt diagnosis, given the median time to diagnosis in patients discharged to rehabilitation facility (21 days) compared to those discharged at home (37 days) [32].

Three patients of the group with postoperative SSI (*n* = 16) eventually developed periprosthetic joint infection and were thus treated with surgical debridement and revision surgery. The incidence of joint osteoarthritis was higher in women compared to men, and the majority of patients were aged above 65 years. No statistically significant difference was noted between gender and type of procedure for the development of SSIs.

26.8% of enrolled patients were transfused with blood, while two of the patients who intraoperatively received tranexamic acid were finally transfused. These data are confirmed in a previous study by Liu W et al. in 2018 [33]. Even if the results did not reveal any statistically significant difference among patients receiving autologous and heterologous blood transfusion, there was an increased risk for SSI in patients with autologous blood transfusion.

The average anticipated blood loss during THR and TKR is around 1500 ml [34], and taking into account that the majority of patients is aged over 65 years and present comorbidities, the blood loss needs to be substituted. A multidisciplinary approach to the preoperative examination, including laboratory tests [35] and a treatment similar to that employed in trauma patients is important for the clinical outcome in patients over 65 years old [36]. Blood transfusion can be either heterologous (allogenic) or autologous. Both options entail complications [37]. The use of autologous blood transfusion was not associated with an elevated infection risk in TKR, testifying to its safety and efficacy for treating postoperative anaemia [38, 39], whereas patients who received allogenic blood transfusion after TKR or THR showed a significantly higher probability of developing wound and lung infections compared to those not transfused or receiving autologous blood [40]. Even if revision rates due to infection were increased in patients with perioperative allogenic transfusion compared to those without, this was not a significant independent predictive factor of reoperation for suspected infection [41]. Moreover, the extensive use of tranexamic is beneficial for preventing blood loss and transfusion without increasing the risk of periprosthetic infection [42, 43].

The results of this study are in alignment with previously published data about the relationship between operative time, hospitalisation length, and the risk of postoperative SSI [44, 45]. The incidence of SSI and PJI was 2.1% and 1.4%, respectively, for operations lasting more than 90 minutes and 1.1% and 0.7% for those lasting under 60 minutes, respectively [45]. Furthermore, as shown in previous studies [46], the length of postoperative hospitalisation affects the prevalence of SSIs and PJIs.

The presence of thyroid gland disease or recurrent infections, especially of the urinary tract system (UTI), are significant contributors to postoperative SSIs and PJIs [47, 48], as also proven by the results of this study [47, 48]. The presence of UTIs prior to joint replacement was a predisposing factor for SSIs and PJIs [48], whereas postoperative UTIs increased the risk for the development of superficial and deep SSIs [49]. Interestingly, no recent study has proven a correlation between recurrent urinary traction infections and SSIs.

Although diabetes mellitus without adequate perioperative glycaemic control has been associated with an increased risk for SSIs, as observed in 20% of diabetic patients following THR or TKR [50], no association was revealed from the data under examination. This is probably a result of the closely monitored diabetic patients being enrolled in the study [51]. It is well established that one of the benefits of sufficient preoperative glycaemic control is the prevention of detrimental postoperative outcomes, such as SSI following joint arthroplasty [52]. Finally, the extracted data demonstrated no association between tobacco consumption and the development of wound infection, confirming the results of many researchers reporting that the risk of PJIs is not affected by smoking status but by other risk factors affecting microvascular functionality, immunological response, and tissue oxygenation, such as coronary heart disease, hypertension, or respiratory diseases [53, 54].

### 4.1. Strengths and Limitations

This study is the first to examine the association between common predictive risk factors including the preoperative quality of life level index and the risk of SSIs, with a long follow-up period of the enrolled subjects (the last follow-up was five years after the procedure). Nevertheless, our study has several limitations. First, the small sample size of the enrolled subjects is a significant drawback. The sample of patients and controls was representative of the whole area population in terms of age, sex, BMI, and OA radiological severity. Moreover, our study includes data from a single large university center and thus a significant number of patients with low severity OA stages may be not selected. In contrast with multicenter studies, subjects with rare clinical conditions were not included. Third, seasonal variations may not have been taken into consideration.

## 5. Conclusion

The aim of our study is to reveal the independent risk factors for SSIs after arthroplasty, which are summarised in longer surgical interventions, hypothyroidism, and recurrent urinary tract infections. Although no statistically significant correlation between periprosthetic infection or SSIs and autologous or heterologous blood transfusion was observed, an effective way to correct anaemia and its consequences is to prevent preoperative anaemia by using erythropoietin, taking into account that the administration of this agent has certain implications. Finally, a clinically relevant point, which is different from other studies, is the association between poor preoperative QoL and the possibility of postoperative periprosthetic SSI. This could be explained by the elevation of preinflammatory cytokines when a patient is in chronic stress condition, as in chronic pain.

## Figures and Tables

**Table 1:** Baseline demographic characteristics of the patients enrolled in our study.

Patient characteristics		*N* ^*∗*^	*N*1^^*∗∗*^^		*N*_2_^^*∗∗∗*^^	*N*%
Sex	Male	18	10		8	32.1
	Female	38	27		11	67.9

Age in years	≥65	38	29		9	67.9
	<65	18	8		10	32.1

Smoking	Yes	7	2	5		12.5

Comorbidity	Coronal artery disease	6	3	3		10.7
	Diabetes mellitus	13	9	4		23.2
	Hypertension	37	26	11		66.1
	Thyroid disease	19	11	8		33.9
	Cancer	5	2	3		8.9
	Recurrent infections of the respiratory system or urinary tract	4	3	1		7.1
	Kidney disease	5	2	3		8.9
	Blood disease	2	2	0		3.6

Comorbidities	Chronic diseases	10	6	4		17.9
	1-2	31	22	9		55.4
	3+	15	9	6		26.8

^^*∗*^^Total number of subjects. ^^*∗∗*^^Number of subjects treated with TKR. ^^*∗∗∗*^^Number of subjects treated with THR.

**Table 2:** Preoperative and postoperative laboratory findings of the included population.

Parameters	Preop values (median value ± SD)	Postop values (median value ± SD)	Laboratory values at hospital discharge	% differences	*p*
Haematocrit (%)	41.7 ± 3.9	36.8 ± 4.1	32.5 ± 4.4	−21.8	<0.001
Haemoglobin (g/dl)	13.4 ± 1.3	11.8 ± 1.3	10.5 ± 1.4	−21.2	<0.001
Creatinine (mg/dl)	0.94 ± 0.23	1.23 ± 0.37	0.99 ± 0.30	+6.3	0.265
Urea (mg/dl)	41.8 ± 17.5	38.2 ± 17.0	36.9 ± 15.6	−7	0.020

**Table 3:** Laboratory findings at preoperative, postoperative, and hospital discharge periods among the TKR and THR groups.

Parameters	Preop values (median value ± SD)	Postop values (median value ± SD)	Laboratory values at hospital discharge
Haematocrit (%) (TKR group)	41.6 ± 8.8	37 ± 4.1	31.5 ± 5.3
Haemoglobin (g/dl) (TKR group)	13.6 ± 1.1	11.8 ± 0.4	11.7 ± 6.3
Creatinine (mg/dl) (TKR group)	1.07 ± 1.1	1.22 ± 0.35	0.99 ± 0.30
Urea (mg/dl) TKR	41.1 ± 17.1	38.6 ± 16.9	36.3 ± 15.4
Haematocrit (%) (THR group)	41.8 ± 6.4	37.0 ± 2.0	33.2 ± 6.4
Haemoglobin (g/dl) (THR group)	13.45 ± 0.1	11.9 ± 0.8	11.5 ± 2
Creatinine (mg/dl) (THR group)	1.08 ± 0.12	1.4 ± 0	1.0 ± 0.1
Urea (mg/dl) THR (THR group)	41.6 ± 21.2	38.2 ± 14.1	36.8 ± 5.7

**Table 4:** Surgical parameters of the included population.

	*N* ^^*∗*^^	TKR (*N*1^^*∗∗*^^, %)	THR (*N*2^^*∗∗∗*^^, %)
Cases	56	37 (66.1)	19 (33.9)
Regional anaesthesia	38 (69.1)	28 (77.8)	10 (52.6)
General anaesthesia	17 (30.9)	8 (22.2)	9 (47.4)
Polymethylmethacrylate (PMMA)	38 (67.9)	37 (100.0)	1 (5.3)
Autologous blood transfusion	13 (23.2)	6 (16.2)	7 (36.8)
Tramadol (painkiller)	21 (37.5)	15 (40.5)	6 (31.6)
Fever	5 (9.3)	4 (11.1)	1 (5.6)
Operation time (in minutes, expressed as mean value ± standard deviation)	91 ± 26	81 ± 22	107 ± 23
Days in hospital (expressed as mean value ± standard deviation)	5.2 ± 1.2	4.9 ± 1.2	5.7 ± 1.0

^^*∗*^^Total number of subjects. ^^*∗∗*^^Number of subjects treated with TKR. ^^*∗∗∗*^^Number of subjects treated with THR.

**Table 5:** Risk factors associated with SSI.

Parameters	SSI				Non-SSI		*p* values
	*N* ^^*∗*^^	*N*_1_^^*∗∗*^^	*N*_2_^^*∗∗∗*^^	Rate (%)	*N*	Rate (%)	
Gender							0.9285
Males	6	3	3	37.5	12	30	
Females	10	7	3	62.5	18	45	
Age groups							0.0011
>65 years	11	11	0	68.7	27	67.5	
≤65 years	5	3	2	31.25	13	32.5	
Perioperative factors							
Type of intervention (cemented)	11	9	2	68.75	15	37.5	0.4731
Autologous blood transfusion	4	3	1	25	5	12.5	0.8427
Duration of intervention (>90 min)	8	7	1	50	6	15	0.0063
Hospitalisation days (>4 days)	13	12	1	81.25	16	40	0.0033
Chronic disease/comorbidities (total)							0.855
Coronary artery disease	2	1	1	12.5	4	10	0.4984
Diabetes mellitus	3	3	0	18.75	10	25	0.2339
Hypertension	12	11	1	75	25	62.5	0.3306
Thyroid gland disease	6	5	1	37.5	13	32.5	0.0329
Cancer	2	1	1	12.5	3	7.5	0.5066
Recurrent infections	3	2	1	18.75	1	2.5	0.0345 < 0.05
Renal disease	1	1	0	6.25	4	10	0.1419
Blood disease	0	1	0	0	2	5	0.3667
Smoking	1	1	0	6.25	6	15	0.3754

^^*∗*^^Total number of subjects. ^^*∗∗*^^Number of subjects treated with TKR. ^^*∗∗∗*^^Number of subjects treated with THR.

**Table 6:** The KOOS and HOOS-quality of life assessment scales of the 56 patients who had THR or TKR.

Parameters	Total scores	KOOS scale for total knee replacement (expressed as median value ± SD)	HOOS scale for total hip replacement (expressed as median value ± SD)	*p* values
Pain	42.3 ± 15.9	43.4 ± 13.3	40.3 ± 20.4	0.319
Symptoms	49.0 ± 18.4	50.7 ± 16.1	45.8 ± 22.5	0.306
Function	39.5 ± 13.9	41.6 ± 12.2	35.4 ± 16.2	0.121
Quality of life	22.1 ± 12.8	23.7 ± 13.1	19.1 ± 11.9	0.200

**Table 7:** Assessment of patient's QOL using the KOOS and HOOS scores based on or not on the development of SSI.

Parameters	QoL scores without SSI (expressed as median value ± SD)	QoL scores in TKR without SSI (expressed as median value ± SD)	QoL scores in THR without SSI (expressed as median value ± SD)	QoL scores with SSI (expressed as median value ± SD)	QoL scores with SSI in TKR (expressed as median value ± SD)	QoL scores with SSI in THR (expressed as median value ± SD)	*p* values
Pain	45.7 ± 16.3	58 ± 13.8	63.3 ± 5.8	33.9 ± 11.7	52.3 ± 14.6	49.6 ± 1.4	0.009
Symptoms	48.4 ± 18.8	54.0 ± 15.4	59.3 ± 16.9	50.4 ± 18.1	40.1 ± 10.4	51 ± 5.65	0.891
Function	41.2 ± 13.6	47.5 ± 10.1	55 ± 4.6	35.2 ± 14.0	41 ± 4.6	47.4 ± 3.5	0.197
Quality of life	22.5 ± 13.2	28.3 ± 12.9	34.5 ± 13.4	21.1 ± 12.0	27.3 ± 14.4	29.5 ± 3.4	0.861

## Data Availability

The data are not publicly available and have been kept on a secure server as per ethics guidelines. The data used to support this study are available from the corresponding author upon request.
